# Stress-activated brain-gut circuits disrupt intestinal barrier integrity and social behaviour

**DOI:** 10.21203/rs.3.rs-3459170/v1

**Published:** 2023-10-27

**Authors:** Scott Russo, Kenny Chan, Long Li, Lyonna Parise, Flurin Cathomas, Katherine LeClair, Yusuke Shimo, Hsiao-yun Lin, Romain Durand-de Cuttoli, Antonio Aubry, Johana Alvarez, Tory Drescher, Aya Osman, Chongzhen Yuan, Rachel Fisher-Foye, Gabrielle Price, Yasemin Schmitt, Manuella Kaster, Glaucia C. Furtado, Sergio Lira, Jun Wang, Wenfei Han, Ivan de Araujo

**Affiliations:** Icahn School of Medicine at Mount Sinai; Icahn School of Medicine at Mount Sinai; Icahn School of Medicine at Mount Sinai; Icahn School of Medicine at Mount Sinai; Icahn School of Medicine at Mount Sinai; Icahn School of Medicine at Mount Sinai; Icahn School of Medicine at Mount Sinai; Icahn School of Medicine at Mount Sinai; Icahn School of Medicine at Mount Sinai; Icahn School of Medicine at Mount Sinai; Icahn School of Medicine at Mount Sinai; Icahn School of Medicine at Mount Sinai; Icahn School of Medicine at Mount Sinai; Icahn School of Medicine at Mount Sinai; Icahn School of Medicine at Mount Sinai; Icahn School of Medicine at Mount Sinai; Icahn School of Medicine at Mount Sinai; The Icahn School of Medicine at Mount Sinai; Icahn School of Medicine at Mount Sinai; Mt. Sinai medical school; Mt. Sinai

## Abstract

Chronic stress underlies the etiology of both major depressive disorder (MDD) and irritable bowel syndrome (IBS), two highly prevalent and debilitating conditions with high rates of co-morbidity. However, it is not fully understood how the brain and gut bi-directionally communicate during stress to impact intestinal homeostasis and stress-relevant behaviours. Using the chronic social defeat stress (CSDS) model, we find that stressed mice display greater intestinal permeability and circulating levels of the endotoxin lipopolysaccharide (LPS) compared to unstressed control (CON) mice. Interestingly, the microbiota in the colon also exhibit elevated LPS biosynthesis gene expression following CSDS. Additionally, CSDS triggers an increase in pro-inflammatory colonic IFNγ^+^ Th1 cells and a decrease in IL4^+^ Th2 cells compared to CON mice, and this gut inflammation contributes to stress-induced intestinal barrier permeability and social avoidance behaviour. We next investigated the role of enteric neurons and identified that noradrenergic dopamine beta-hydroxylase (DBH)^+^ neurons in the colon are activated by CSDS, and that their ablation protects against gut pathophysiology and disturbances in social behaviour. Retrograde tracing from the colon identified a population of corticotropin-releasing hormone-expressing (CRH^+^) neurons in the paraventricular nucleus of the hypothalamus (PVH) that innervate the colon and are activated by stress. Chemogenetically activating these PVH CRH^+^ neurons is sufficient to induce gut inflammation, barrier permeability, and social avoidance behaviour, while inhibiting these cells prevents these effects following exposure to CSDS. Thus, we define a stress-activated brain-to-gut circuit that confers colonic inflammation, leading to impaired intestinal barrier function, and consequent behavioural deficits.

Chronic psychosocial stress is a major risk factor for neuropsychiatric disorders including major depressive disorder (MDD), as well as functional gastrointestinal disorders such as irritable bowel syndrome (IBS), two of the most prevalent and debilitating illnesses that show high rates of co-morbidity^1^. Potentially underlying this co-morbidity is the recent finding that chronic stress elicits low-grade inflammation, which is associated with the severity of both MDD and IBS symptoms^3,4^. Importantly, both of these conditions are multifactorial disorders with limited treatment options; thus, there is a need to identify biological changes associated with their pathogenesis. Moreover, increasing evidence suggests that the gut-brain axis, or connections between the central (CNS) and enteric nervous systems (ENS), contributes to the etiology of MDD and IBS^5,6^. However, the mechanisms by which psychological states such as chronic stress influence gut pathophysiology, including inflammation and permeability, remain poorly understood. Further, the impact of gut inflammation on gut-to-brain signaling and stress-relevant behaviours is largely unexplored.

The intestinal barrier has gained attention for its role in inflammatory diseases, as it regulates the translocation of pro-inflammatory antigens and microbial compounds from the gut lumen into circulation, and is home to 70% of the body’s immune cells^7^. An increase in circulating bacterial toxins – or endotoxaemia – is hypothesized to initiate and propagate systemic inflammation by activating innate immune responses during chronic inflammatory conditions^8^. Importantly, there is evidence that the intestinal barrier becomes compromised in patients with MDD or IBS^9–11^. However, the mechanisms by which this occurs and the consequences on neuropsychiatric symptoms including behaviour remain largely unknown. In this study, we use a mouse model of chronic psychosocial stress to uncover a pathway through which the CNS, ENS, and immune system communicate to trigger gut inflammation, barrier permeability, and social avoidance behaviour.

## Chronic social stress promotes intestinal permeability and endotoxaemia

**Figure 1:**
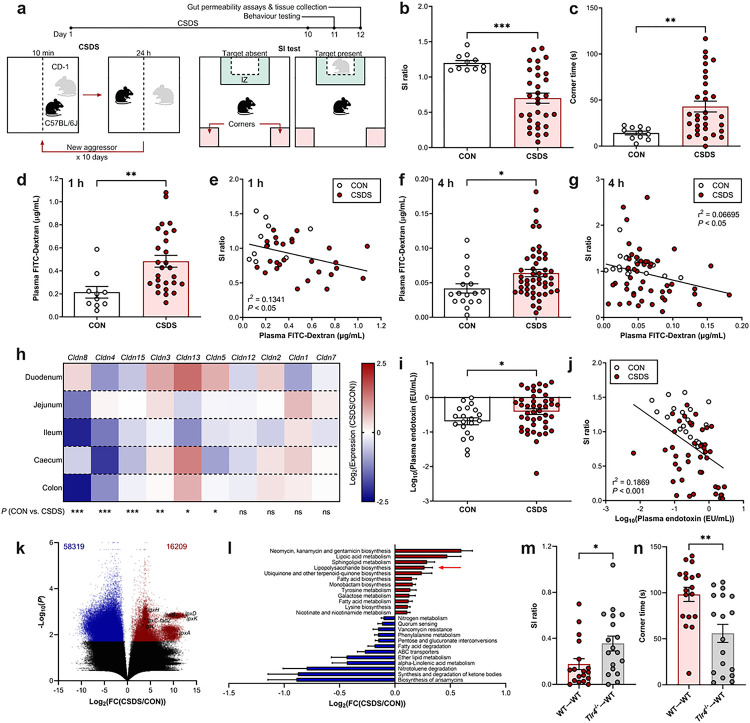
Chronic social stress triggers gut permeability and endotoxaemia. **a,** Experimental timeline and schematic of CSDS and social interaction (SI) test. **b, c,** SI ratio defined as time spent in the interaction zone (IZ) while a social target is present divided by the time spent in the IZ when the target is absent (**b**) and time spent in corners while the target is present (c) during SI test (*n* = 11–31). **d–g,** Intestinal permeability assessed by plasma FITC levels 1 h (*n* = 11–27) (**d, e**) or 4 h (*n* = 17–48) (**f, g**) following oral gavage with 4 kDa FITC-Dextran, and correlated with SI ratio. **h,** Fold change (CSDS/CON) in claudin family tight junction gene expression throughout the intestinal tract (*n* = 6–17). **i, j,** Plasma endotoxin concentrations after CSDS, and correlation with SI ratio (*n* = 20–44). **k, l,** Functional metagenomic profiling of colon contents following CSDS. Volcano plot showing significantly upregulated (red) and downregulated (blue) genes (adjusted *P* value, q < 0.2) with select genes in the LPS biosynthesis pathway highlighted (**k**) and select KEGG pathway enrichment (**l**). Arrow indicates LPS biosynthesis pathway (*n* = 8). **m, n,** SI ratio (**m**) and time spent in corners (**n**) in mice lacking haematopoietic *Tlr4* (*Tlr4*^−/−^→WT) or wild-type controls (WT→WT) exposed to CSDS (*n* = 18). Data expressed as means ± s.e.m. **b-d, f, i, m, n,** Two-tailed unpaired t test. **e, g, j,** Linear regression with Pearson’s correlation coefficient. **h,** Two-way ANOVA with Bonferroni’s multiple comparisons test. **k, l,** Kruskal-Wallis H test . *P < 0.05, **P < 0.01, ***P < 0.001.

Using the chronic social defeat stress (CSDS) model (Fig. 1a), which induces social avoidance behaviour in both male (Fig. 1b, c) and female experimental mice (**Extended Data Fig. 1a**, **b**), we first tested if animals exposed to chronic psychosocial stress display increased intestinal permeability. After orally gavaging mice with 4 kDa FITC-dextran, which does not normally cross an intact intestinal epithelium, we assessed FITC fluorescence in plasma 1 or 4 h later, corresponding to gastrointestinal transit time from the stomach to the small or large intestine, respectively^12^. At both time points, male and female mice that underwent CSDS showed significantly greater gut permeability compared to unstressed control (CON) mice, with plasma FITC concentrations correlating with social avoidance behaviour (Fig. 1d–g, **Extended Data Fig. 1c**, **d**). Importantly, when gut permeability was measured before the 10 day CSDS protocol, there were no pre-existing differences in permeability before stress exposure that predicted consequent social avoidance behaviour. In fact, the majority of mice that underwent CSDS displayed increased gut permeability after the 10 day paradigm (**Extended Data Fig. 1e**–**h**), indicating that a leaky gut is indeed a consequence of chronic stress. Additionally, several claudins, which form tight junctions between epithelial cells, such as *Cldn4, Cldn5, Cldn8,* and *Cldn15,* were transcriptionally downregulated throughout the intestine following CSDS compared to CON mice (Fig. 1h). Further, there was a marked reduction in goblet cell number, but not cell size, in the colons of mice after CSDS (**Extended Data Fig. 1i**–**k**), suggesting a loss of mucous, which normally protects the intestinal epithelium against pathogenic invasion^13^. These results demonstrate that chronic stress compromises intestinal barrier integrity.

As a potential consequence of a damaged intestinal barrier, we analysed circulating levels of lipopolysaccharide (LPS), a component of the gram-negative bacterial outer membrane that elicits pro-inflammatory host responses upon recognition by its receptor toll-like receptor 4 (TLR4)^14^. After CSDS, stressed mice had significantly higher plasma LPS levels than CON mice, with circulating LPS concentrations negatively correlating with social interaction behaviour (Fig. 1i, j). Of note, we found no correlation between the degree of wounding acquired during CSDS and plasma LPS levels (**Extended Data Fig. 1l**); we therefore speculated that stress-induced endotoxaemia originates from gut translocation rather than exogenous infection. We next performed functional metagenomic analysis of colon luminal contents from stressed and CON mice. First, we identified that CSDS-exposed mice had a distinct microbiome compared to CON mice, characterized by a shift in beta diversity and a reduced Firmicutes/Bacteroidetes ratio (**Extended Data Fig. 2a**–**e**). Next, while the majority of differentially expressed microbial genes regulated by stress were downregulated in colon contents following CSDS, genes involved in the LPS biosynthesis pathway were significantly enriched (Fig. 1k, l, **Extended Data Fig. 2f**, **Supplementary Table 1**), suggesting that CSDS-induced endotoxaemia may be a consequence of both increased barrier leakiness and LPS production by gut bacteria. Hypothesizing that gut permeability and endotoxaemia could initiate systemic inflammation contributing to social avoidance behaviour, we depleted TLR4 from haematopoietic cells via bone marrow transplantation to restrict gene depletion to peripheral immune cells, then subjected these chimeric mice to CSDS (**Extended Data Fig. 3a**–**d**). Interestingly, we found that mice lacking haematopoietic TLR4 (*Tlr4*^−/−^→WT) were partially protected against social avoidance triggered by CSDS, compared to mice transplanted with wild-type bone marrow (WT→WT) (Fig. 1m, n). Taken together, these results indicate that chronic stress disrupts the intestinal barrier, allowing LPS translocation into circulation, which impacts social behaviour.

## Colonic inflammation contributes to stress-evoked gut permeability

**Figure 2:**
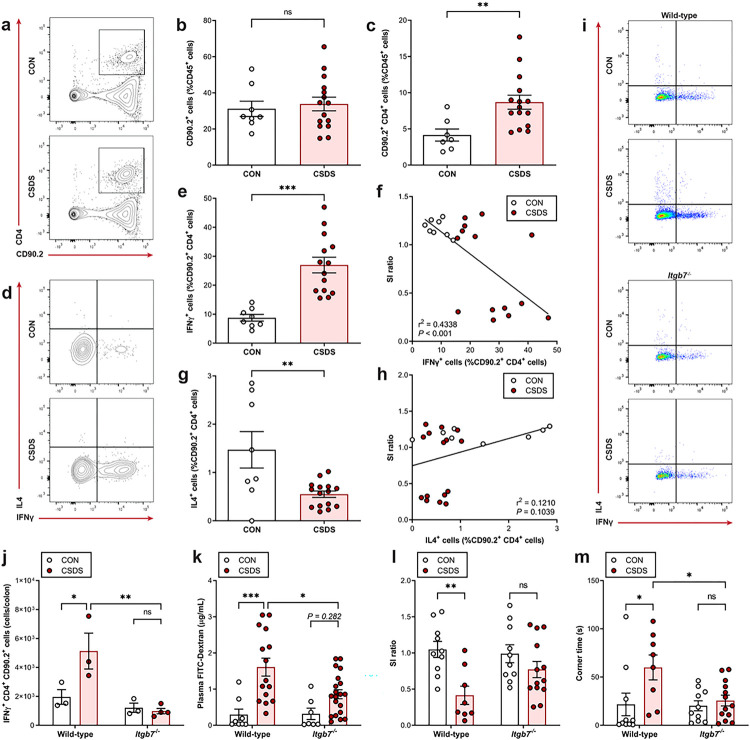
Stress-induced colonic inflammation contributes to intestinal permeability and social avoidance. **a–h,** Lamina propria lymphocytes were isolated from colon tissue following CSDS and analysed by flow cytometry. Representative flow cytometry plots of CD90.2^+^ and CD4^+^ cells (%CD45^+^ cells) (**a**) or IFNγ^+^ and IL4^+^ cells (%CD90.2^+^ CD4^+^ cells) (**d**). Quantification of total T cells (**b**), CD4^+^ T cells (c), Th1 cells (**e**) correlated with SI ratio (**f**), and Th2 cells (**g**) correlated with SI ratio (**h**) (*n* = 7–15). **i–m,** ITGβ7-deficient or wild-type littermate control mice were subjected to CSDS. Representative flow cytometry plots of IFNγ^+^ and IL4^+^ CD4^+^ T cells from the colon (**i**), quantification of Th1 cells (*n* = 3–4) (**j**), gut permeability 4 h following oral gavage with 4 kDa FITC-Dextran (*n* = 7–20) (**k**), SI ratio (*n* = 8–13) (**l**), and time spent in corners during the SI test (*n* = 8–13). Data expressed as means ± s.e.m. **b, c, e, g,** Two-tailed unpaired t test. **f, h,** Linear regression with Pearson’s correlation coefficient. **j–m,** Two-way ANOVA with Tukey’s multiple comparisons test. *P < 0.05, **P < 0.01, ***P < 0.001, ns. Not significant.

We next investigated how stress could impact intestinal permeability, hypothesizing that pro-inflammatory polarization of enteric immune cells breaks down the gut barrier, as seen in conditions such as inflammatory bowel disease (IBD), obesity, and intestinal ischaemia^15,16^. Focusing on the colon, where most gut bacteria are found, we isolated lamina propria lymphocytes from the colons of both male and female mice under CSDS and CON conditions, and assessed specific T cell subpopulations by flow cytometry. In both sexes, while there were no differences in total T cell frequencies between CON and CSDS mice, there were significantly greater numbers of CD4^+^ helper T cells, and notably, increased pro-inflammatory IFNγ^+^ Th1 cells in mice exposed to CSDS, while IL4^+^ Th2 cells were reduced (Fig. 2a–h, **Extended Data Fig. 4a**–**d**). Moreover, we did not observe increases in IL17A^+^ Th17 cells or innate immune cells often associated with colon inflammation, such as monocytes, neutrophils, and dendritic cells (**Extended Data Fig. 4e**–**m**). CSDS therefore triggers Th1-mediated colonic inflammation.

To determine the contribution of this inflammation to stress-induced intestinal permeability and social behaviour deficits, we used mice lacking integrin β7 (ITGβ7), a transmembrane protein necessary for gut homing of immune cells^17^. After confirming that the majority of enteric leukocytes express ITGβ7, and that *Itgb7*^−/−^ mice had reduced total leukocytes, B cells, and T cells in the colon (**Extended Data Fig. 5a**–**e**) compared to wild-type mice, we put *Itgb7*^−/−^ and littermate wild-type control mice through CSDS, and found that ITGβ7-deficiency ameliorates stress-induced colon inflammation (Fig. 2i, j, **Extended Data Fig. 5f**). In addition, *Itgb7*^−/−^ mice were partially resistant to gut permeability, and social avoidance triggered by CSDS (Fig. 2k–m), indicating that intestinal immune cells play a role in these stress-linked phenotypes.

## Noradrenergic enteric neurons influence gut inflammation and barrier permeability

**Figure 3:**
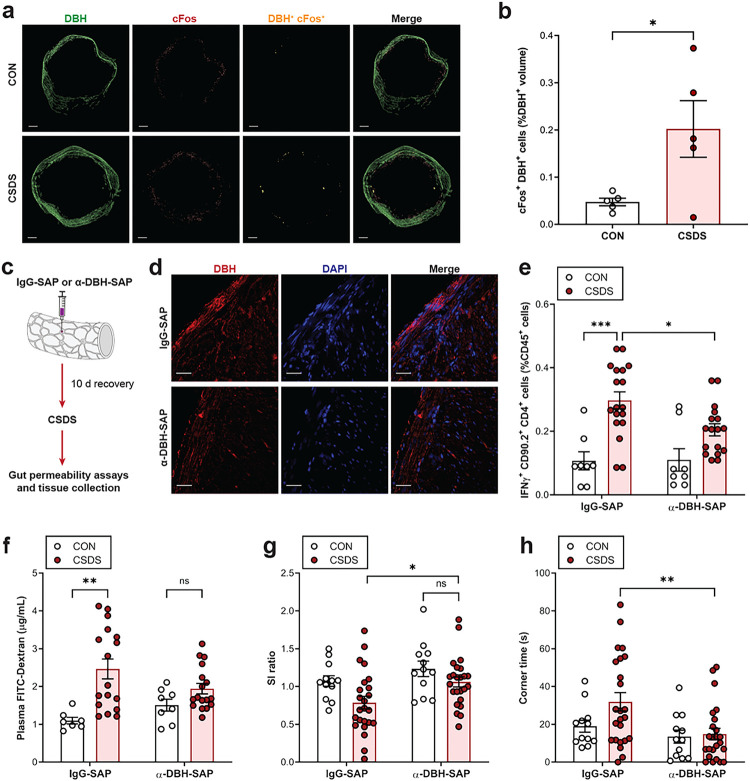
Noradrenergic enteric neuron activation promotes intestinal pathophysiology during stress. **a, b,** Colon tissue was collected from CON or CSDS mice, and activation of DBH^+^ neurons was assessed using whole-tissue clearing and imaging. Representative images (scale bar = 1000 μm) (**a**) and quantification (**b**) showing percentage of DBH^+^ cells expressing cFos (*n* = 5). **c–h,** Noradrenergic neurons were ablated by injecting anti-DBH (α-DBH) or control IgG conjugated to saporin (SAP) into the colon wall. Following 10 d of recovery, mice were subjected to CSDS (**c**). Representative images showing DBH expression in the colon (scale bar = 50 μm) (**d**). Quantification of Th1 cells (%CD45^+^ cells) (**e**) from the colon lamina propria (*n* = 8–18). Gut permeability 4 h following oral gavage with 4 kDa FITC-Dextran (*n* = 7–17) (**f**). SI ratio (**g**) and time spent in corners (**h**) during SI test (*n* = 12–24). Data expressed as means ± s.e.m. **b,** Two-tailed unpaired t test. **e–h,** Two-way ANOVA with Tukey’s multiple comparisons test. *P < 0.05, **P < 0.01, ***P < 0.001, ns. Not significant.

As chronic stress heightens sympathetic tone from the autonomic nervous system (ANS), in conjunction with the colon receiving dense noradrenergic innervation from neurons in the myenteric and submucosal plexus^18–20^, we examined if noradrenergic enteric neurons are involved in gut inflammation and barrier permeability during CSDS. We first assessed if noradrenergic neurons in the myenteric and submucosal plexus become activated by stress. Using iDISCO+ whole-tissue clearing and staining of colon samples from CON and CSDS mice, we found that in the CSDS group, enteric neurons expressing dopamine beta-hydroxylase (DBH), a marker for noradrenergic neurons, show significantly elevated levels of cFos, a molecular marker of neuronal activity (Fig. 3a, b). To understand if these neurons contribute to stress-induced gut pathophysiology, we injected saporin (SAP) conjugated to anti-DBH antibodies throughout the wall of the colon, to effectively ablate DBH^+^ neurons selectively in the colon (Fig. 3c, d). Upon exposure to CSDS, we observed that anti-DBH-SAP blunted the stress-evoked increase in Th1 cells in the colon compared to mice that received control infusions of IgG-SAP (Fig. 3e). Moreover, colonic DBH^+^ cell depletion dampened CSDS-induced gut permeability (Fig. 3f) and social avoidance behaviour (Fig. 3g, h). These experiments demonstrate that noradrenergic enteric neurons become activated by psychosocial stress and regulate colonic inflammation and barrier permeability.

## Stress-induced gut pathophysiology is regulated by the CNS

**Figure 4:**
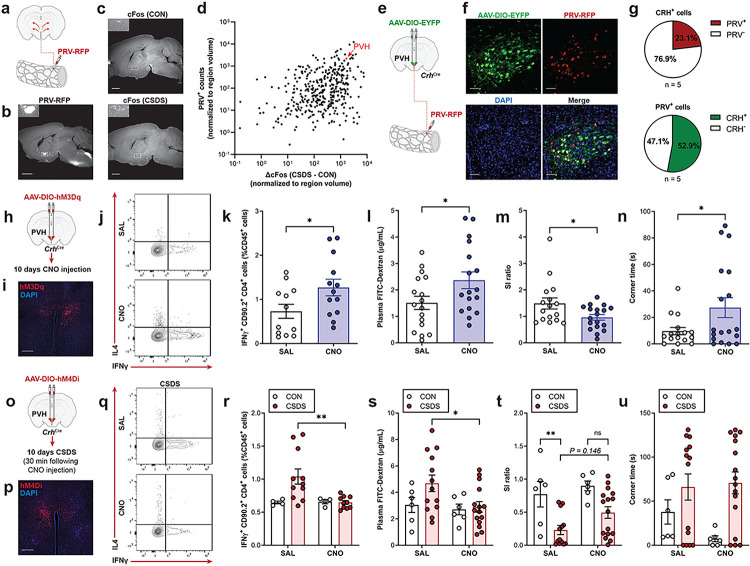
Stress-mediated gut inflammation and barrier permeability are centrally regulated. **a–d,** PRV-RFP was injected into the colon for polysynaptic retrograde tracing (**a**). Representative sagittal section following whole-brain clearing and imaging showing RFP expression 5 d after PRV-RFP infection (scale bar = 150 μm) (**b**) or cFos expression in CON or CSDS mice (scale bar = 150 μm). Inset shows PVH (**c**). ClearMap was used to measure PRV^+^ cells or increase in cFos^+^ cells following CSDS per brain region (each dot represents one brain region; arrow highlights PVH) (*n* = 4–10) (**d**). **e–g,** *Crh*^Cre^ mice were administered Cre-dependent AAV-DIO-EYFP in the PVH and PRV-RFP in the colon (**e**). Representative PVH images (scale bar = 50 μm) (**f**) and quantification of EYFP^+^ RFP^+^ cells (*n* = 5) (**g**). **h–n,** Excitatory DREADD hM3Dq was expressed in CRH^+^ cells in the PVH, then activated with 1.0 mg/kg CNO i.p. for 10 d (**h**). Representative PVH section showing viral expression (scale bar = 200 μm) (**i**). Representative flow cytometry plots (**j**) and quantification (**k**) of Th1 cells from the colon (*n* = 12–13). Intestinal permeability assessed by plasma FITC levels 4 h after oral gavage with 4 kDa FITC-Dextran (*n* = 17) (**l**). SI ratio (**m**) and time spent in corners (**n**) during SI test (*n* = 16–18). **o–u,** Inhibitory DREADD hM4Di was expressed in CRH^+^ cells in the PVH, then stimulated with 1.0 mg/kg CNO i.p. 30 min before CSDS for 10 d (**o**). Representative PVH section showing viral expression (scale bar = 200 μm) (**p**). Representative flow cytometry plots (**q**) and quantification (**r**) of Th1 cells from the colon (*n* = 4–11). Intestinal permeability assessed by plasma FITC levels 4 h after oral gavage with 4 kDa FITC-Dextran (*n* = 6–15) (**s**). SI ratio (**t**) and time spent in corners (**u**) during SI test (*n* = 6–16). Data expressed as means ± s.e.m. **k–n,** Two-tailed unpaired t test. **r–u,** Two-way ANOVA with Tukey’s multiple comparisons test. *P < 0.05, **P < 0.01, ns. Not significant.

Mechanistically, we aimed to identify brain regions that become activated by stress that can convey signals to the ENS to influence intestinal inflammation and barrier function. To accomplish this, we first injected pseudorabies virus carrying RFP (PRV-RFP) into the colon as a polysynaptic retrograde tracer, then performed whole-brain clearing with iDISCO+ and light sheet imaging to label CNS regions that project to the colon (Fig. 4a, b). In parallel, we assessed neuronal activity by staining cFos in whole brains following CSDS (Fig. 4c), and generated a comprehensive map of stress-activated gut-innervating brain regions (Fig. 4d, **Supplementary Table 2**). We identified the paraventricular nucleus of the hypothalamus (PVH) as a brain region with both strong connectivity to the colon and significantly increased cFos expression following CSDS. Given that the PVH is implicated in regulating immunological processes in peripheral tissues, such as the spleen and bone marrow, as well as homeostatic gastrointestinal functions including motility^21,22^; we chose to investigate this region further. Our next objective was to characterise neuronal subpopulations in the PVH that innervate the colon. We hypothesized that corticotropin-releasing hormone (CRH)-expressing neurons could be involved, as CRH knockout ameliorates inflammation during experimental colitis, and intracerebroventricular CRH administration can mimic stress-induced colonic dysmotility^23,24^. To assess if PVH CRH^+^ neurons project to the gut, we delivered Cre-dependent adeno-associated virus expressing EYFP (AAV-DIO-EYFP) into the PVH of *Crh*^*Cre*^ mice, then three weeks later, injected PRV-RFP into the colons of the same mice (Fig. 4e). Co-localization of EYFP and RFP revealed that approximately 50% of all gut-innervating PVH neurons express CRH (Fig. 4f, g), confirming that these neurons are structurally connected to the colon.

To test whether PVH CRH^+^ neurons can control enteric immunity and barrier function, we expressed the excitatory designer receptor exclusively activated by designer drugs (DREADD) hM3Dq in the PVH of *Crh*^*Cre*^ mice. Similar to a previously established method to induce stress responses by chronically activating CRH^+^ cells^25^, we performed chemogenetic activation of PVH CRH^+^ neurons by administering clozapine N-oxide (CNO) once daily for 10 consecutive days, to mimic conditions during the 10 day CSDS paradigm (Fig. 4h, i). Interestingly, independent of stress, chronic PVH CRH^+^ neuron stimulation was sufficient to induce low-level colon inflammation and gut barrier permeability, and diminish social interaction behaviour (Fig. 4j–n). Importantly, CNO administration alone did not impact gut physiology or social behaviour in the absence of PVH DREADD expression (**Extended Data Fig. 6a**–**e**). As PVH CRH^+^ neurons may influence peripheral immune responses and behaviour through neuroendocrine mechanisms via the HPA axis^26^, we administered metyrapone to block corticosterone synthesis during chemogenetic excitation of PVH CRH^+^ cells and found no effects on gut barrier permeability (**Extended Data Fig. 7 a, b**). Moreover, HPA inhibition was found to worsen social avoidance behaviour induced by PVH CRH^+^ neuron activation (**Extended Data Fig. 7c**, **d**); thus, central regulation of gut pathophysiology and associated behavioural deficits may be exerted through direct neural connections via the ANS rather than the HPA axis. Lastly, we explored whether silencing PVH CRH^+^ neurons could mitigate CSDS-induced gut and behavioural phenotypes using the inhibitory DREADD hM4Di (Fig. 4o, p). Inhibition of PVH CRH^+^ neurons throughout CSDS partially prevented stress-induced gut inflammation, barrier permeability and social avoidance (Fig. 4q–u). Collectively, we identified that CRH^+^ neurons in the PVH are directly linked to the colon and can regulate enteric physiology during stress.

## Discussion

Here, we describe a pathway in which psychosocial stress activates CRH^+^ neurons in the PVH, which relay signals to the ENS. Noradrenergic enteric neurons then trigger Th1-mediated colonic inflammation, which contributes to intestinal barrier permeability and endotoxaemia. Consequently, circulating endotoxins are detected by haematopoietic TLR4 to promote social avoidance behaviour. Our findings are consistent with previous reports showing that activating PVH CRH^+^ neurons can re-capitulate inflammatory leukocyte dynamics in the bone marrow and spleen, similar to what is observed following stress exposure^27,28^, and that depleting CRH receptor type 1 (CRFR1) from the PVH prevents CSDS-induced anxiety-like behaviour^29^. Further, CRH has been implicated in inflammation associated with experimental colitis^23^. Interestingly, a recent study found that chronic restraint stress exacerbates dextran sodium sulphate (DSS)-induced colitis through a mechanism dependent on the HPA axis, enteric glia, and colonic monocytes^30^. Together with our findings, this suggests that depending on disease context, chronic stress may elicit colonic inflammation through either the peripheral nervous system or HPA axis, and involve enteric neurons and glia, along with colonic T cells and monocytes. In the case of CSDS, our data suggest that PVH CRH^+^ neurons control intestinal permeability and inflammation through enteric neurons via the ANS, but not necessarily through HPA activation or stress hormones.

Recent single-cell and single-nucleus sequencing datasets have confirmed the presence of neurons expressing DBH and noradrenaline in the colon^19,20^. Our results showing that colonic DBH^+^ neurons become hyperactive during stress suggest that there might be enteric neuron subsets implicated in psychiatric and inflammatory disorder co-morbidity. Although there is evidence that noradrenaline acts on β2-adrenergic receptors (β2AR) on CD4^+^ T cells to influence their differentiation and cytokine expression, opposing outcomes have been reported depending on timing of activation. While noradrenaline can suppress IFNγ production by Th1 cells, β2AR stimulation on naïve CD4^+^ T cells results in greater IFNγ expression upon differentiation into Th1 cells^31^. Thus, we hypothesize that colonic noradrenergic neurons act on naïve CD4^+^ T cells to provoke gut inflammation during stress.

Moreover, we speculate that Th1-mediated inflammation causes gut permeability as IFNγ is known to directly disrupt intestinal epithelial barrier formation, permitting bacterial translocation^32^. However, it is possible that additional colonic immune cells are involved, as other pro-inflammatory cytokines such as TNFα have been demonstrated to downregulate epithelial tight junction proteins during IBD^33^. While measurements of endotoxaemia during chronic stress and psychiatric disorders have yielded mixed results, faecal samples from patients with MDD or anxiety show enrichment for LPS biosynthesis pathway genes, consistent with our findings in mice^9,34^. In addition, whole-body TLR4 depletion in mice is protective against stress-induced social avoidance and learned helplessness^35,36^. Our experiments using bone marrow chimeric mice specifically implicate haematopoietic TLR4 in this process. Notably, peripheral blood cells from patients with MDD or from mice susceptible to CSDS also display heightened sensitivity to TLR4 stimulation^37–39^.

The stress-activated brain-gut circuits described in this study represent potentially novel targets for the treatment of stress-related psychiatric disorders, in a tissue that is more accessible for pharmacological intervention than the brain. As antidepressant drugs are predominantly administered orally, it is possible that existing therapeutic options may exert effects through the ENS and intestinal immune system. Notably, antidepressants are effective in treating IBS, although whether the mechanisms of action are local or top-down through the CNS, remain to be fully elucidated^40,41^. It is therefore critical for future work to further dissect how the bi-directional communication between the brain and gut, which involves the immune system, becomes dysregulated under pathological conditions, and to identify new treatment targets to prevent or reverse such effects.

## Methods

### Animals.

Wild-type (C57BL/6J), *Esr1*^Cre^ (B6N.129S6(Cg)-*Esr1*^*tm1.1(cre)And*^/J; strain: 017911), *Tlr4*^−/−^ (B6(Cg)-*Tlr4*^*tm1.2Karp*^/J; strain: 029015), CD45.1 (B6.SJL-*Ptprc*^*a*^ *Pepc*^*b*^/BoyJ; strain: 002014), *Itgb7*^−/−^ (C57BL/6-*Itgb7*^*tm1Cgn*^/J; strain: 002965), and *Crh*^Cre^ (B6(Cg)-*Crh*^*tm1(cre)Zjh*^/J; strain: 012704) mice were purchased from The Jackson Laboratory. CD-1 (Crl:CD1(ICR); strain: 022) mice were purchased from Charles River Laboratories. *Tlr4*^−/−^ and *Itgb7*^−/−^ mice were mated with wild-type mice, and F1 heterozygous offspring were subsequently mated to produce littermate *Tlr4*^+/+^ and *Tlr4*^−/−^, or *Itgb7*^+/+^ and *Itgb7*^−/−^ offspring for experimentation. Mice were allowed to habituate to the vivarium for at least one week before usage. Animals were maintained on a 12 h-12 h light-dark cycle (lights on from 07:00 to 19:00) at 22 °C with *ad libitum* access to food and water, and group-housed until experimentation. Before behavioural testing, mice were acclimated to the testing room for at least 1 h. All procedures were performed in accordance with the National Institutes of Health Guide for the Care and Use of Laboratory Animals and the Icahn School of Medicine at Mount Sinai Institutional Animal Care and Use Committee (IACUC) (protocols IACUC-2014–0081, IACUC-2017–0241, and LA10–00266 to S.J.R.).

### Bone marrow transplantation.

To generate chimeric mice lacking TLR4 in haematopoietic cells, 6 week-old male CD45.1 recipient mice were lethally irradiated (11 Gy, in two 5.5 Gy doses, 4 h apart) using an X-rad 320 Irradiator (Precision X-Ray, Madison, CT). Bone marrow cells were then isolated from the femurs and tibia of 10–12 week-old male littermate wild-type or *Tlr4*^−/−^ donor mice, passed through a 70 μm cell strainer, and re-suspended at a concentration of 1 × 10^7^ cells/mL in PBS. Recipient mice were then anaesthetized with isoflurane and a total of 1 × 10^6^ donor cells were injected retro-orbitally. Chimeric mice were given neomycin trisulphate (0.2% w/v in drinking water; N1876, Sigma-Aldrich, St. Louis, MO) for 10 days, then allowed to recover for an additional 3 weeks before experimentation.

### *In vivo* interventions and procedures

#### Chronic social defeat stress.

Chronic social defeat stress (CSDS) in male^2^ and female^42^ mice was performed as previously reported. For CSDS in male mice, 4–6 month-old male CD-1 retired breeder mice were screened for aggressive behaviour for three consecutive days, and non-aggressive mice were excluded. Aggressive CD-1 mice were housed on one side of a perforated Plexiglas partition in a hamster cage (26.7 × 48.3 × 15.2 cm) at least two days before CSDS. Experimental mice were then subjected to an encounter with an aggressive CD-1 mouse for 10 min (5 min for chimeric or denervated mice) per day, then transferred to the opposite side of the partition to allow for sensory but not physical interaction for the remainder of the day. This procedure was repeated for 10 consecutive days with a new aggressor each day. Unstressed control mice were pair-housed across a perforated Plexiglas partition. For CSDS in female mice, aggressors were generated by first crossing homozygous *Esr1*^Cre^ mice with CD-1 mice, then bilaterally injecting Cre-dependent AAV2-hSyn-DIO-hM3D(Gq)-mCherry (44361-AAV2, Addgene, Watertown, MA) into the ventrolateral subdivision of the ventromedial hypothalamus in the heterozygous F1 offspring. Aggressive behaviour was subsequently elicited by intraperitoneally (i.p.) injecting mice with 1.0 mg/kg clozapine-*N*-oxide (CNO; 4936, Tocris, Bristol, United Kingdom) to activate ERα^+^ cells 30 min before CSDS. Experimental mice were then subjected to a 5 min physical encounter once per day with a new aggressor for each day for 10 consecutive days. Following the final day of CSDS, experimental mice were single-housed (male) or group-housed (female). All mice were carefully examined for wounding throughout CSDS experiments, with mice with excess wounding according to previously established criteria excluded^43^.

#### Social interaction test.

Social interaction (SI) testing was performed as previously described^2^, 24 h after the final day of CSDS. Following a 1 h habituation period under red light conditions in the behavioural suite, mice were allowed to freely explore a Plexiglas arena (45 × 45 × 45 cm, Nationwide Plastics, Arlington, TX) with an empty wire enclosure on one end for 2.5 min. Next, experimental mice were removed from the arena, and a novel social target mouse was placed into the wire enclosure. Experimental mice were then returned to the arena for an additional 2.5 min. Locomotor activity was tracked and recorded using a Basler GenICam (acA1300–60, Basler, Ahrensberg, Germany) coupled to Noldus Ethovision version 11.0 (Noldus Information Technology, Leesburg, VA). SI ratio was calculated as the ratio of time spent in the 24 × 14 cm interaction zone around the wire enclosure in the presence of a social target divided by time spent in the absence of a social target. Corner time was calculated as the cumulative time spent in the two 9 × 9 cm corner zones opposite of the wire enclosure in the presence of a social target.

#### FITC-Dextran gut permeability assay.

FITC-Dextran with an average molecular weight of 3000 – 5000 Da (FD4, Sigma-Aldrich) was prepared at a concentration of 120 mg/mL in mouse drinking water. Mice were fasted for at least 4 h, then orally gavaged with 600 mg/kg FITC-Dextran using 20 G × 38 mm flexible gavage needles (FTP-20–38, Instech Laboratories, Plymouth Meeting, PA). At least one mouse per experiment was gavaged with drinking water alone for background subtraction. After 1 or 4 h, trunk blood or blood from the submandibular vein was collected into heparin-coated microcentrifuge tubes (41.1503.150, Sarstedt, Newtown, NC), then centrifuged at 375 × *g* for 10 min. Plasma was separated into new tubes then diluted 1 in 10 in PBS. Diluted plasma or FITC-Dextran standards in PBS were added to black 96-well plates, and plasma FITC-Dextran concentrations were measured on a SpectraMax Gemini XS fluorescence microplate reader (Molecular Devices, San Jose, CA) using an excitation wavelength of 490 nm and emission wavelength of 520 nm. Mean fluorescence intensity in plasma from water-gavaged mice was subtracted as background fluorescence from all samples, and plasma FITC-Dextran concentrations were calculated by interpolating fluorescence values against a standard curve.

#### Local colonic denervation.

Anti-DBH-SAP (IT-03, Advanced Targeting Systems, Carlsbad, CA) or control IgG-SAP (IT-18, Advanced Targeting Systems) were prepared at a final concentration of 0.7 mg/mL in sterile saline with 0.005% Fast Green FCF dye (F7252, Sigma-Aldrich) for visualization. Seven week-old male C57BL/6J mice were anaesthetized with isoflurane, and the surgical site was shaved and sterilized with iodine and alcohol swabs. An approximately 1 cm lower abdominal midline incision was made with sterile surgical scissors, and the caecum and colon were carefully extracted from the peritoneal cavity onto a saline-moistened gauze pad using sterile forceps and cotton swabs. Under a dissection microscope, anti-DBH-SAP or IgG-SAP was injected into the colonic mucosa using a NanoFil Sub-microliter Injection System (World Precision Instruments, Sarasota, FL) using 36 G beveled needles (NF36BV-2, World Precision Instruments), connected to a Legato 180 syringe pump (KD Scientific, Holliston, MA). Approximately 120 nL of anti-DBH-SAP or IgG-SAP was injected at each injection site at a rate of 40 nL/s throughout the length of the colon. The caecum and colon were then returned to the peritoneal cavity, and the abdominal muscles and skin were sutured. Mice were given 10 days to recover post-surgery before experimentation.

#### Retrograde tracing.

Mice were anaesthetized with isoflurane. The surgical site was prepared, and colon was extracted as described above. Under a dissection microscope, approximately 120 nL of PRV-RFP (1.9 × 10^9^ vg/mL, PRV-614, Center for Neuroanatomy with Neurotropic Viruses, Pittsburgh, PA) was injected at a single site in the proximal colon at a rate of 40 nL/s. The caecum and colon were then returned to the peritoneal cavity, and the abdominal muscles and skin were sutured. Five days after PRV infection, brain tissue was collected for immunohistochemistry or iDISCO+ clearing and staining.

#### Stereotaxic surgery.

Mice were anaesthetized with an i.p. injection of 100 mg/kg ketamine HCl and 10 mg/kg xylazine then positioned on a stereotaxic instrument (David Kopf Instruments, Tujunga, CA). In the PVH (from bregma: AP −1.0 mm; ML ±0.5 mm; DV −4.5 mm, with an angle of 5°), 0.7 μL of AAV9-hSyn-DIO-EYFP (2.0 × 10^12^ vg/mL, 50457-AAV9, Addgene), AAV8-hSyn-DIO-hM3D(Gq)-mCherry (2.0 × 10^12^ vg/mL, 44361-AAV8, Addgene), AAV9-hSyn-DIO-hM4D(Gi)-mCherry (2.0 × 10^12^ vg/mL, 44362-AAV9, Addgene), or AAV9-hSyn-DIO-mCherry (2.0 × 10^12^ vg/mL, 50459-AAV9, Addgene) were bilaterally infused using 33 G Hamilton needles over 7 min, with the needle left in place for 5 min following the injection. In the VMHvl (from bregma: AP: −1.5 mm; ML ±0.7 mm, DV: −5.7 mm), 0.7 μL of AAV2-hSyn-DIO-hM3D(Gq)-mCherry (2.0 × 10^12^ vg/mL, 44361-AAV2, Addgene) were bilaterally infused using 33 G Hamilton needles over 7 min, with the needle left in place for 5 min following the injection.

#### Chemogenetic manipulation.

To activate ERα^+^ cells in aggressors for female CSDS, 1.0 mg/kg CNO was given i.p. 30 min before CSDS. For chronic activation of PVH CRH^+^ neurons, 1.0 mg/kg CNO was given intraperitoneally once per day for 10 consecutive days. For chronic inhibition of PVH CRH^+^ neurons, 1.0 mg/kg CNO was given i.p. once per day, 30 min before CSDS, for 10 consecutive days. Control mice were injected with saline i.p.

#### HPA axis inhibition.

The corticosterone synthesis inhibitor metyrapone (BML-EI256–0200, Enzo, Farmingdale, NY) was used to inhibit the HPA axis as previously described^44^. Metyrapone was dissolved in saline to a concentration of 10 mg/mL. 30 min before experimentation, mice were injected i.p. with a dose of 100 mg/kg metyrapone or saline alone as control.

### Cells

#### Lamina propria lymphocyte isolation.

Whole colon tissue was dissected into ice-cold PBS + 2 U/mL penicillin + 2 μg/mL streptomycin (15140–122, ThermoFisher Scientific, Waltham, MA). Mesenteric fat was removed, tissue was cut open longitudinally, colon contents were gently removed with a cotton swab, and tissue was cut into approximately 0.5 cm fragments. Tissue fragments were washed in PBS + penicillin/streptomycin, then mucous was removed by incubating in PBS + 1 mM DTT + 10 mM HEPES (25–060-CI, Corning, Corning, NY) at room temperature for 10 min with agitation. After washing again with PBS + penicillin/streptomycin, tissue fragments were incubated in PBS + 5.2 mM EDTA (15575–038, ThermoFisher Scientific) + 10 mM HEPES at room temperature for 13 min with agitation to dissociate epithelial cells. Samples were then washed five times with PBS + 2% FBS (10438–026, ThermoFisher Scientific) + penicillin/streptomycin, then transferred to microcentrifuge tubes containing RPMI (11875–085, ThermoFisher Scientific), and finely minced. Minced tissue samples were then digested in RPMI + 10% FBS + penicillin/streptomycin + 1 mg/mL collagenase type IV (17104–019, ThermoFisher Scientific) + 0.5 mg/mL dispase II (D4693, Sigma-Aldrich) + 100 μg/mL DNAse I (DN25, Sigma-Aldrich) at 37 °C for 45 min with shaking. Samples were then passed through a 70 μm cell strainer then centrifuged at 500 × *g* for 8 min. Supernatant was decanted and cell pellets were re-suspended in 44% Percoll (17089101, Cytiva, Marlborough, MA), then carefully layered over 66% Percoll. Percoll density gradients were centrifuged at 1100 × *g* at 4 °C for 25 min with low acceleration and braking, then lamina propria lymphocytes were collected from the layer at the 44%/66% Percoll interphase into new conical tubes. For myeloid cell isolation, density gradient separation was not performed. Cells were washed in PBS + 2% FBS and centrifuged at 500 × *g* for 8 min. Supernatant was decanted, and cells were re-suspended in PBS + 2% FBS until counting, stimulation, or staining.

#### *Ex vivo* lymphocyte stimulation.

For analysis of intracellular cytokines by flow cytometry, lamina propria lymphocytes were re-suspended at a concentration of 1 × 10^6^ cells/mL in RPMI + 10% FBS + 1 U/mL penicillin + 1 μg/mL streptomycin + 55 μM 2-mercaptoethanol (21985–023, ThermoFisher Scientific) + 1X non-essential amino acids (M7145, Sigma-Aldrich) + eBioscience cell stimulation cocktail (00-4970-93, ThermoFisher Scientific) + BD GolgiStop protein transport inhibitor (554724, BD Biosciences, Franklin Lakes, NJ). Samples were incubated at 37 °C for 4 h, then centrifuged at 2300 × *g* for 30 s to pellet cells. Supernatant was discarded and cells were washed in FACS buffer (PBS + 0.5% BSA + 2 mM EDTA) before staining for flow cytometry.

### Molecular assays

#### Endotoxin measurement.

Plasma endotoxin concentrations were analysed using the PYROGENT^™^−5000 Kinetic Turbidimetric limulus amebocyte lysate (LAL) Assay (N383, Lonza, Morristown, NJ) according to the manufacturer’s instructions. Following CSDS, trunk blood was collected into heparin-coated microcentrifuge tubes, then centrifuged at 375 × *g* for 10 min. Plasma was separated into new tubes and stored at −80 °C until analysis. For the LAL assay, plasma samples were diluted 1 in 10 in PBS then heat-inactivated at 75 °C for 5 min. Diluted plasma or endotoxin standards were added to 96-well plates, then incubated at 37 °C for 10 min. Next, reconstituted LAL Reagent was added to each well, and absorbance at 340 nm was measured on a SpectraMax 340PC384 microplate reader (Molecular Devices) every 15 s for 1 h. Mean reaction time was determined as the time after absorbance increased by 0.03 O.D. compared to baseline, and endotoxin concentrations were calculated based on mean reaction time of endotoxin standards.

#### Metagenomic sequencing.

For functional metagenomics experiments, to minimize cage effects, co-housed C57BL/6J mice were randomly assigned to either CON or CSDS groups. CON mice were exposed to bedding and faeces from CD-1 aggressor mice to eliminate potentially confounding microbes introduced by the aggressor during CSDS. Colon contents were collected then flash frozen on dry ice and stored at −80 °C until analysis. Microbial DNA was isolated using the DNeasy PowerSoil Pro Kit (47014, Qiagen, Germantown, MD) according to the manufacturer’s instructions. DNA was fragmented with NEBNext dsDNA Fragmentase (M0348S, New England Biolabs, Ipswich, MA) at 37 °C for 30 min. DNA library was constructed using the TruSeq Nano DNA LT Library Preparation Kit (FC-121-4001, Illumina, San Diego, CA). Metagenomic analysis was performed by LC Sciences (Houston, TX) on a NovaSeq 6000 Sequencing System (Illumina) using 2500 ng of DNA. Raw sequencing reads were processed to obtain valid reads for further analysis. First, sequencing adapters were removed from sequencing reads using cutadapt v1.9. Secondly, low quality reads were trimmed by fqtrim v0.94 using a sliding-window algorithm. Thirdly, reads were aligned to the host genome using bowtie2 v2.2.0 to remove host contamination. Once quality-filtered reads were obtained, they were *de novo* assembled to construct the metagenome for each sample by IDBA-UD v1.1.1. All coding regions of metagenomic contigs were predicted by MetaGeneMark v3.26. Coding region sequences of all samples were clustered by CD-HIT v4.6.1 to obtain unigenes. Unigene abundance for a certain sample was estimated by TPM based on the number of aligned reads by bowtie2 v2.2.0. The lowest common ancestor taxonomy of unigenes was obtained by aligning them against the NCBI NR database by DIAMOND v0.0.14. Similarly, functional gene annotation and pathway mapping was conducted with the Kyoto Encyclopedia of Genes and Genomes (KEGG) database. Based on the taxonomic and functional annotation of unigenes, along with the abundance profile of unigenes, the differential analyses were carried out at each taxonomic or functional or gene-wise level by Fisher’s exact test (non-replicated groups) or Kruskal-Wallis test (replicated groups). Statistical significance was set at *P* < 0.05, q < 0.2.

#### qPCR.

Tissue samples were homogenized in TRIzol reagent (15596018, ThermoFisher Scientific), then RNA was extracted according to manufacturer’s instructions. cDNA was synthesized using qScript cDNA SuperMix (95048, Quantabio, Beverly, MA). qPCR was performed using 20 ng cDNA with pre-designed TaqMan probes (4331182, ThermoFisher Scientific) for *Cldn1* (Mm01342184_m1), *Cldn2* (Mm00516703_s1), *Cldn3* (Mm00515499_s1), *Cldn4* (Mm00515514_s1), *Cldn5* (Mm00727012_s1), *Cldn7* (Mm00516817_m1), *Cldn8* (Mm00516972_s1), *Cldn12* (Mm01316511_m1), *Cldn13* (Mm00491038_s1), *Cldn15* (Mm00517635_m1), and *Tlr4* (Mm00445273_m1). qPCR reactions were run on a QuantStudio 7 Flex real-time PCR system (4485701, Applied Biosystems, Waltham, MA). Data were analysed using the 2^−ΔΔCt^ method, and normalized to housekeeping genes *Abt1* (Mm00803824_m1) and *Hprt* (Mm03024075_m1) (4448484, ThermoFisher Scientific).

#### Histology.

Mice were euthanized with 10% chloral hydrate and transcardially perfused with ice-cold PBS. Colon tissue was extracted and fixed in 10% neutral buffered formalin (HT501128, Sigma-Aldrich) in a ‘Swiss roll’ configuration for 48 h, then paraffin-embedded using a Tissue-Tek VIP E300 Tissue Processor (Sakura, Torrance, CA). Formalin-fixed, paraffin-embedded colon tissue was cut into 5 μm sections, and periodic acid-Schiff (PAS) staining was performed using the PAS Reaction Kit (k047, Poly Scientific R&D Corp., Bay Shore, NY) according to the manufacturer’s instructions to label goblet cells. Briefly, de-paraffinized and rehydrated slides were incubated with 0.5% periodic acid for 5 min, Schiff Reagent for 15 min and Harris haematoxylin for 5 min, then quickly dipped in 0.5% acid alcohol followed by 1% lithium carbonate. Sections were dehydrated in 95% ethanol and cleared with xylene, then mounted onto slides. Images were acquired using a Zeiss Axio Imager.M1 microscope (Zeiss). Goblet cell size and number were analysed using ImageJ (National Institutes of Health, Bethesda, MD).

#### Immunohistochemistry.

Following transcardial PBS perfusion, formalin-fixed, paraffin-embedded colon tissue samples were cut onto slides at a thickness of 5 μm using a microtome, then tissue was de-paraffinized and rehydrated as previously described^45^. Antigen retrieval was performed by heating samples in target retrieval solution (S2367, Agilent, Santa Clara, CA) for 15 min. Sections were then incubated with a blocking buffer (PBS + 10% BSA + 1X Tris + 0.1% Triton) for 30 min. Tissue samples were incubated with primary antibodies against DBH (1:100, ab209487, Abcam, Waltham, MA) overnight at 4 °C, then washed twice in PBS before incubation with Cy5-AffiniPure goat anti-rabbit IgG (H+L) (1:400, 111-175-144, Jackson ImmunoResearch Laboratories, West Grove, PA) for 30 min. Slides were then incubated in PBS with DAPI (1 μg/mL, D9542, Sigma-Aldrich) for 10 min. Samples were washed twice with PBS and mounted onto coverslips with EcoMount (EM897L, Biocare Medical, Pacheco, CA). Images were acquired using a Leica SP8 confocal microscope (Leica Microsystems, Deerfield, IL).

To amplify PRV-RFP signal in brain tissue, mice were euthanized with 10% chloral hydrate then transcardially perfused with ice-cold PBS followed by 4% paraformaldehyde (PFA). Brains were post-fixed in 4% PFA for 12 h at 4 °C. Coronal sections were prepared on a vibratome at a thickness of 50 μm. Brain sections were incubated in blocking solution (PBS + 3% normal donkey serum + 0.3% Triton X-100) for 2 h, then incubated with primary antibodies against RFP (1:800, 600-401-379, Rockland Immunochemicals, Pottstown, PA) for 2 h. Samples were then washed three times with PBS and incubated in donkey anti-rabbit IgG (H+L) secondary antibody, Alexa Fluor 568 (1:1000, A10042, ThermoFisher Scientific) for 2 h. Sections were washed three times with PBS before staining with DAPI (1 μg/mL) for 20 min. Slides were then mounted onto coverslips with EcoMount. Images were acquired using a Zeiss LSM 780 confocal microscope (Zeiss, White Plains, NY).

#### Flow cytometry.

For staining of blood samples, red blood cells were first lysed using BD Pharm Lyse (555899, BD Biosciences) according to manufacturer’s instructions, then washed in FACS buffer. For all samples, cells were blocked using anti-CD16/32 (2.5 μg/mL, BE0307, clone 2.4G2, Bio X Cell, Lebanon, NH), and incubated with Fixable Viability Dye eFluor 780 (1:4000, 65-0865-14, ThermoFisher Scientific) on ice for 30 min, then washed. Cell surface staining was then performed with specified antibodies (**Supplementary Table 4**) on ice for 30 min. For staining of intracellular cytokines, cells were incubated in fixation/permeabilization buffer (554714, BD Biosciences) on ice for 30 min, washed with BD Perm/Wash buffer (554714, BD Biosciences), then stained with specified antibodies (**Supplementary Table 4**) at 4 °C in the dark overnight. For cell counting, CountBright absolute counting beads (C36950, ThermoFisher Scientific) were used. Cells were washed, then re-suspended in FACS buffer before acquisition on a BD LSRFortessa cell analyzer (BD Biosciences). Data were analysed using FlowJo version 10.6.2 (BD Biosciences).

#### iDISCO+ whole-tissue clearing and staining.

The iDISCO+ protocol was adapted from http://www.idisco.info, with incubation times indicated for brain/colon tissues, respectively. Following perfusion with 4% PFA, tissue was post-fixed for 24/3 h in 4% PFA at 4 °C. Tissue was dehydrated in methanol then incubated overnight in 66% dichloromethane (270997, Sigma-Aldrich) + 33% methanol. The next day, samples were washed twice in methanol, and bleached using 5% H_2_O_2_ overnight. Samples were then rehydrated, washed twice in PBS + 0.2% Tween-20 + 10 μg/mL heparin (H3393, Sigma-Aldrich), and incubated in permeabilization solution (PBS + 20% DMSO 0.2% Triton X-100 + 300 mM glycine) at 37 °C for 48/24 h, then in blocking solution (PBS + 10% DMSO + 6% donkey serum (017-000-121, Jackson ImmunoResearch Laboratories) + 0.2% Triton X-100) at 37 °C for 48/24 h. Next, samples were incubated with primary antibodies (**Supplementary Table 5**) in PBS + 0.2% Tween-20 + 10 μg/mL heparin + 5% DMSO + 3% donkey serum at 37 °C for 7/2 days. Tissue was subsequently washed 4 times over the next 48 h, then incubated with secondary antibodies (**Supplementary Table 5**) in PBS + 0.2% Tween-20 + 10 μg/mL heparin + 3% donkey serum at 37 °C for 7/2 days. Samples were again washed 4 times over the following 48/24 h. For tissue clearing, samples were dehydrated in methanol, then incubated in 66% dichloromethane + 33% methanol for 3/1 h, 100% dichloromethane for 15 min twice, and finally in dibenzyl ether (108014, Sigma-Aldrich) until imaging. Images were acquired using a LaVision light sheet microscope (LaVision BioTec, Bielefeld, Germany) with zoom body, using dynamic focus and a step size of 3 – 5 μm. Cleared brains were processed and analysed for cFos^+^ or PRV^+^ counts using ClearMap as previously described^46^. Cleared colon tissue was analysed for overlapping DBH^+^ and cFos^+^ surfaces using Imaris 10.0.1 (Oxford Instruments, Abingdon, UK) with Labkit machine learning pixel classification.

#### Enzyme-linked immunosorbent assay.

Blood was collected from the submandibular vein into heparin-coated microcentrifuge tubes then centrifuged at 375 × *g* for 10 min. Plasma was separated and stored at −80 °C until analysis. Plasma corticosterone was measured using a Corticosterone ELISA Kit (ADI-900-097, Enzo) according to the manufacturer’s instructions.

### Statistics.

Detailed statistical information for each experiment can be found in the corresponding figure legends and **Supplementary Table 3**. Unless otherwise specified, statistics were performed using GraphPad Prism software (GraphPad Software, Boston, MA). Statistical significance was set at *P* < 0.05.

## Data Availability

Metagenomic sequencing data have been deposited into the NIH Sequence Read Archive (SRA) under BioProject ID PRJNA1022140. All other data are available from the corresponding author upon reasonable request.
